# Air Pollution and Birth Weight: New Clues about a Potential Critical Window of Exposure

**DOI:** 10.1289/ehp.123-A242

**Published:** 2015-09-01

**Authors:** Nancy Averett

**Affiliations:** Nancy Averett writes about science and the environment from Cincinnati, OH. Her work has been published in *Pacific Standard*, *Audubon*, *Discover*, *E/The Environmental Magazine*, and a variety of other publications.

Researchers have previously reported associations between exposure to air pollution during pregnancy and decreased birth weights.^1^^,^^2^ However, in any given location there is usually very little variation in air pollutant concentrations over short time periods, barring events such as wildfires and other seasonally influenced sources of pollution. It has therefore been difficult to pinpoint a particular window of time during gestation when an exposed fetus might be particularly susceptible to air pollutants.^3^ In this issue of *EHP*, investigators report findings on birth weight arising from a unique research opportunity: the temporary decline in air pollution during the 47 days comprising the 2008 Beijing Summer Olympic Games and Paralympic Games.^3^

Because Beijing typically has some of the world’s highest levels of air pollution, China was required to reduce local air pollution for the duration of the event as a condition of hosting the Games. After temporarily implementing stricter emissions standards and shutting down nearby industrial polluters, pollutant measurements between June and October 2008 showed reductions of 18–59% compared with pre-Olympics levels. But once the Games were over, air pollution began to rise again.^4^^,^^5^

**Figure:**
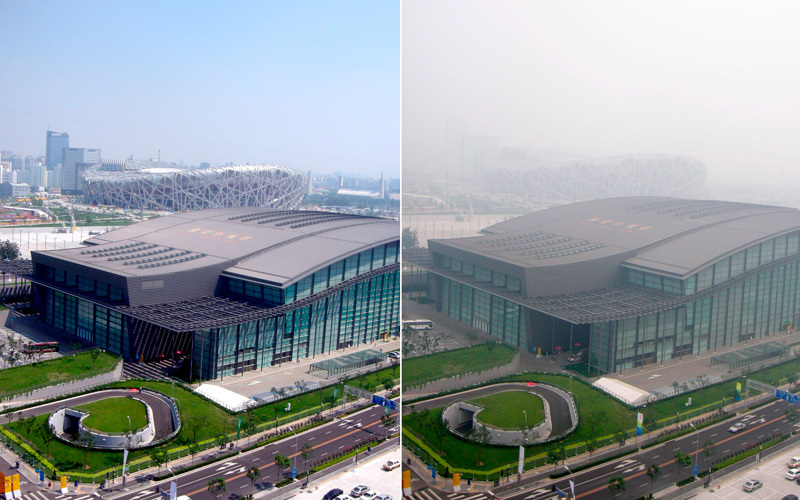
China launched an intensive effort to clean up Beijing’s air for the 2008 Summer Olympics. The drastic measures did achieve noticeable results. However, levels of air pollution were still high, and air quality varied widely from day to day, as illustrated by these photos of Olympic venues taken 24 hours apart in August 2008. © Hand Goedel/epa/Corbis

The current study included two month-by-month analyses. The authors included only term births (37–41 weeks) and births in which the infant was neither large nor small for gestational age.

In the first analysis, the researchers assessed records for 71,803 births at Beijing Obstetrics and Gynecology Hospital to determine which month of each pregnancy fell during the Games (8 August–24 September 2008). They compared the birth weights with those in which the same month of pregnancy occurred in the same calendar period either one year earlier or one year later. They found the strongest association when the eighth month fell during the Games, with babies born in 2008 weighing an average of 23 g (0.8 oz) more than the babies born in 2007 or 2009.^3^

In the second analysis, the team used data on air pollution, temperature, and humidity gathered at a site in central Beijing to calculate average monthly concentrations of PM_2.5_, CO, NO_2_ and SO_2_ during each month of pregnancy. They then looked at 32,506 birth records in which women were pregnant for at least one full month during the Games. They found that interquartile increases in the pollutants during the eighth month of pregnancy were associated with reductions of 17–34 g in birth weight, depending on the pollutant. They found no such associations for increased air pollution during other months of pregnancy.^3^

Lead author David Rich, a public health sciences professor at the University of Rochester Medical Center, says it will take further study to figure out why the eighth month may be a critical time for pregnant women and air pollution exposure. “It could be that the early months are important—if pollution levels went down in the first or second month, it could have a beneficial effect on the baby’s growth—but then the pollution levels went back up after the Olympic Games, so any improvement in birth weight may be ‘washed away.’” He says another possibility is that since late pregnancy is when the fetus grows most rapidly, air pollutant exposure during this time period may affect some biologic mechanism—perhaps the placenta’s ability to deliver nutrients—that impedes fetal growth.

A 2014 meta-analysis of previous studies showed that increased concentrations of PM_10_ and PM_2.5_ across the span of a pregnancy were associated with increases in the risk of low birth weight and decreased birth weight among term births.^6^ However, a previous natural experiment, which used data from the closure of a large Utah Valley steel mill between August 1986 and September 1987, did not find a difference in birth weights between babies born before, during, or after the closure.^7^

David Savitz, a professor of epidemiology at Brown University School of Public Health, says he found the new results more persuasive than previous studies looking at the impact of air pollution in reducing birth weights. “Some people are already convinced that it’s a known fact that pollution [lowers birth weights],” he says. “I’m not among them. I’m pretty skeptical about it.” But while this study is far from the final word on the issue, he says it “really adds a significant amount of weight to the evidence in support of an effect on birth weight.” Savitz was not involved in the study.

Lyndsey Darrow, an assistant professor of epidemiology at Emory University who also was not involved in the study, says she would like to see a similar study but with a longer duration of reduced air pollution. “Ideally, future complementary studies could take advantage of other natural experiments to investigate the cumulative impact of reducing air pollution over the full course of pregnancy,” she says, adding, “Of course, such natural experiments are hard to come by.”

## References

[1] Pedersen M Ambient air pollution and low birthweight: a European cohort study (ESCAPE). Lancet Respir Med 1 9 695 704 2013; 10.1016/S2213-2600(13)70192-9 24429273

[2] Dadvand P Maternal exposure to particulate air pollution and term birth weight: a multi-country evaluation of effect and heterogeneity. Environ Health Perspect 121 3 367 373 2013; 10.1289/ehp.1205575 PMC3621183 23384584

[3] Rich DQ Differences in birth weight associated with the 2008 Beijing Olympic air pollution reduction: results from a natural experiment. Environ Health Perspect 123 9 880 888 2015; 10.1289/ehp.1408795 25919693 PMC4559955

[4] Zhang J Cardiorespiratory biomarker responses in healthy young adults to drastic air quality changes surrounding the 2008 Beijing Olympics. Res Rep Health Eff Inst 174 5 174 2013; PMID:23646463 PMC4086245

[5] Rich DQ Association between changes in air pollution levels during the Beijing Olympics and biomarkers of inflammation and thrombosis in healthy young adults. JAMA 307 19 2068 2078 2012; 10.1001/jama.2012.3488 22665106 PMC4049319

[6] Fleischer NL Outdoor air pollution, preterm birth, and low birth weight: analysis of the World Health Organization global survey on maternal and perinatal health. Environ Health Perspect 122 4 425 430 2014; 10.1289/ehp.1306837 24508912 PMC3984219

[7] Parker JD Preterm birth after the Utah Valley Steel Mill closure: a natural experiment. Epidemiology 19 6 820 823 2008; 10.1097/EDE.0b013e3181883d5d 18854706

